# Intraperitoneal metastasis of ovarian cancer: new insights on resident macrophages in the peritoneal cavity

**DOI:** 10.3389/fimmu.2023.1104694

**Published:** 2023-04-25

**Authors:** Taito Miyamoto, Brennah Murphy, Nan Zhang

**Affiliations:** Immunology, Metastasis & Microenvironment Program, Ellen and Ronald Caplan Cancer Center, The Wistar Institute, Philadelphia, PA, United States

**Keywords:** ovarian cancer, intraperitoneal metastasis, resident macrophages, peritoneal cavity, peritoneal fluid, peritoneum, omentum

## Abstract

Ovarian cancer metastasis occurs primarily in the peritoneal cavity. Orchestration of cancer cells with various cell types, particularly macrophages, in the peritoneal cavity creates a metastasis-favorable environment. In the past decade, macrophage heterogeneities in different organs as well as their diverse roles in tumor settings have been an emerging field. This review highlights the unique microenvironment of the peritoneal cavity, consisting of the peritoneal fluid, peritoneum, and omentum, as well as their own resident macrophage populations. Contributions of resident macrophages in ovarian cancer metastasis are summarized; potential therapeutic strategies by targeting such cells are discussed. A better understanding of the immunological microenvironment in the peritoneal cavity will provide a stepping-stone to new strategies for developing macrophage-based therapies and is a key step toward the unattainable eradication of intraperitoneal metastasis of ovarian cancer.

## Introduction

According to Globocan’s 2020 projections, the incidence rate of ovarian cancer is expected to climb from ~300,000 new cases to ~430,000 new cases worldwide by 2040 (an increase of nearly 37%), with mortality rates also projected to increase by more than 50% (1). Currently, more than 200,000 women die annually from ovarian cancer, making it the second most deadly gynecological cancer (1). Because effective screening methods for early detection have not yet been established (2), patients are often found to have advanced disease that has spread outside the ovaries at the time of diagnosis (3). Although maximum efforts should be made for tumor reduction because complete resection at the primary surgery leads to a good prognosis, complete resection is often difficult due to the spread of the disease (4). Despite advancements in molecular targeted therapies (such as the recent introduction of poly (ADP-ribose) polymerase (PARP) inhibitors and anti-vascular endothelial growth factor (VEGF) monoclonal antibodies), recurrence is inevitable for most patients and the disease eventually becomes resistant to treatment (5, 6). Efficacies of immune checkpoint therapies remain limited in ovarian cancer, which is why immunotherapies have not yet become a standard treatment option (7–10).

Ovarian cancer cases with peritoneal metastasis are very common. More than 75% of patients show intraperitoneal metastases at the time of their first surgery (11) and 75% of recurrent disease is intraperitoneal (12). Because the epithelium of the ovary or the fallopian tube (where ovarian cancer originates) is exposed in the abdominal cavity with no anatomic barriers (13), ovarian cancer cells easily detach from the primary tumor and enter the peritoneal fluid. These cells disseminate throughout the entire peritoneal cavity but preferentially metastasize to the peritoneum and omentum. Here, cancer cells thrive in a favorable tumor microenvironment for survival, engraftment, and development through various interactions with stromal cells (14, 15). Unfortunately, eradication of intra-abdominal lesions in ovarian cancer is an unresolved issue and the efficacy of intraperitoneal chemotherapy and hyperthermic intraperitoneal chemotherapy (HIPEC) is presently inconclusive (16, 17).

Macrophages in the peritoneal cavity play an important role in shaping the tumor microenvironment in ovarian cancer metastasis (18). It has been known for more than half a century that macrophages are abundant in the peritoneal fluid (19). It is now clear that multiple macrophage populations with unique characteristics are present not only in the peritoneal fluid, but also in the peritoneum and omentum in the steady state. These are called tissue-resident macrophages. Elegant lineage-tracing studies in mice in the past decade reveal that tissue-resident macrophages generally have two distinct origins, embryonic precursors prenatally and bone marrow precursors postnatally (i.e., monocyte-derived). The ratios between these two fractions vary across tissue types and are regulated by tissue-specific signals (20–25). Macrophages of different origins can exhibit hardwired differences that may not be as “plastic” as we previously thought (26). Macrophages in tumors (tumor-associated macrophages; TAMs) consist of different proportions of these resident fractions which are present before tumor formation and newly-infiltrated monocyte-derived fraction which come during tumor progression. Importantly, TAMs with different origins have been suggested to play different roles in tumor progression (27). Notably, embryonically-derived resident macrophages in the peritoneal fluid, peritoneum, and omentum have been individually shown to have tumor-promoting role in ovarian cancer (24, 25, 28). Therefore, targeting these cells may be of therapeutic interest for the development of novel anti-cancer immunotherapies.

Here, we provide an overview of the unique microenvironment and resident macrophage populations within the peritoneal fluid, peritoneum, and omentum, as well as their roles in ovarian cancer progression in mice and humans. Detailed knowledge of the intraperitoneal environment, including the origin-based diversity of macrophages, will deepen our understanding about the intraperitoneal metastasis of ovarian cancer. Furthermore, it will help overcome the limited efficacy of macrophage-targeted cancer therapy in clinical settings due to the complexity of macrophage origins, plasticity, and intra-tumor heterogeneity (29, 30), and help toward controlling the ovarian cancer progression in the peritoneal cavity.

## The unique intraperitoneal environment and its contribution to ovarian cancer progression

**Figure 1:**
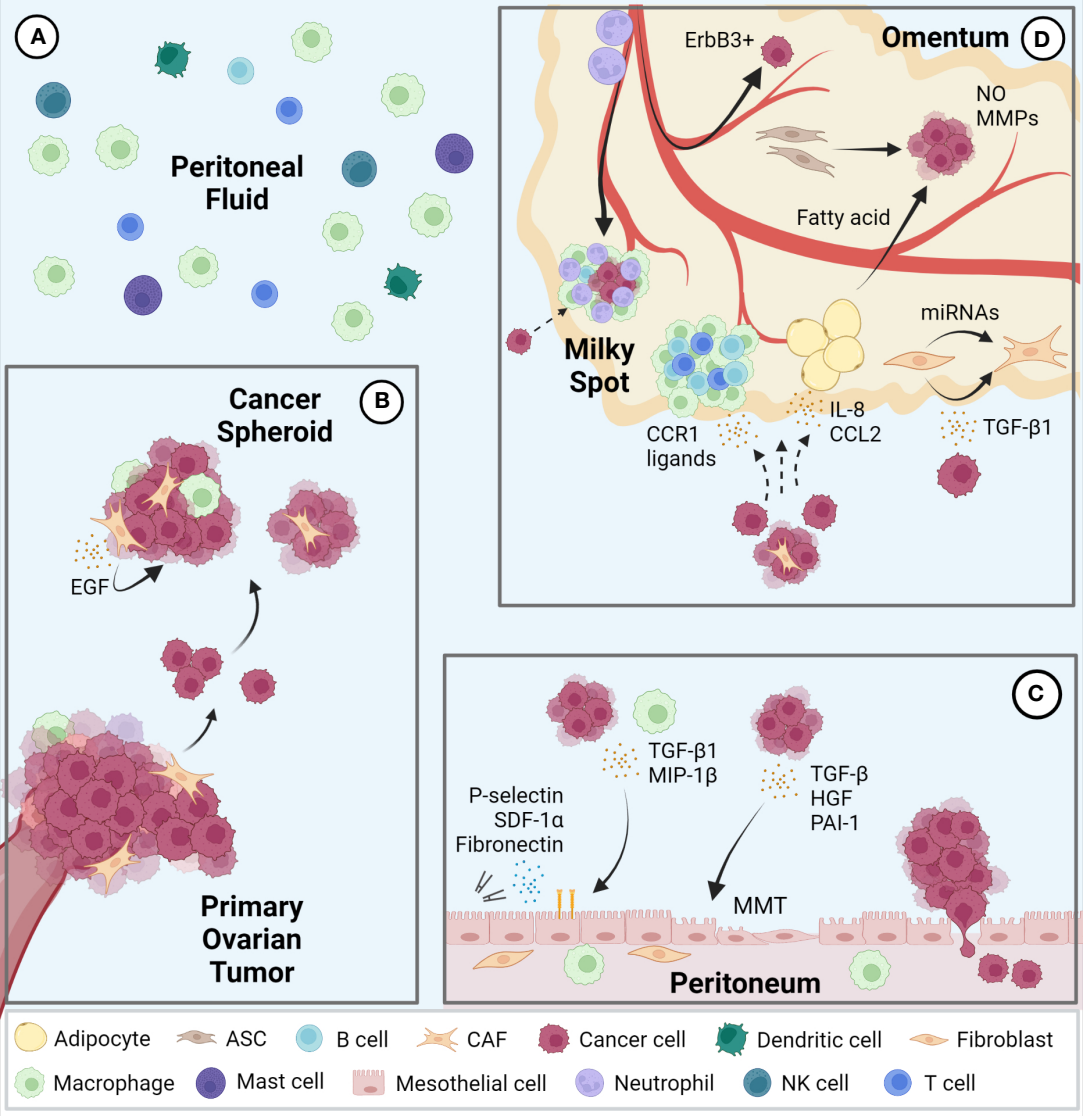
Intraperitoneal metastasis of ovarian cancer. **(A)** Immune cell populations represented in the peritoneal fluid; macrophages represent the largest population followed by T cells, dendritic cells, mast cells, NK cells, and B cells. **(B)** Ovarian cancer cells metastasize via detaching from the primary tumor to form multicellular spheroids containing various stromal cells in the peritoneal fluid and seed in pro-tumor secondary sites including the peritoneum **(C)** and omentum **(D)**. EGF, epidermal growth factor; TGF, transforming growth factor; MIP, macrophage inflammatory protein; MMT, mesothelial-to-mesenchymal transition; SDF, stromal cell-derived factor; hepatocyte growth factor (HGF); PAI, plasminogen activator inhibitor; CCR, C-C motif chemokine receptor; CCL, C-C motif chemokine ligand; NO, nitric oxide; MMP, matrix metalloproteinase; CAF, cancer associated fibroblasts; ASC, adipose-derived stromal cells.

The peritoneal cavity contains serous exudate with various components such as steroid hormones, cytokines, and growth factors at steady state (31, 32). The peritoneal fluid volume is 5-20 mL in humans, which varies widely depending on the physiological condition. For example, in females, this volume changes during the estrus cycle and reaches the maximal level after ovulation (31). Macrophages are the most abundant immune cell population in peritoneal fluid, followed by smaller populations of T cells, dendritic cells, mast cells, NK cells, and B cells (33, 34) (**Figure 1A**). During ovarian cancer metastasis, cancer cells detach from the primary tumor as single cells or clusters that form multicellular spheroids containing other cellular components such as macrophages and fibroblasts. Epidermal growth factor (EGF) secreted from these cells in spheroids promotes cancer cell growth and survival (**Figure 1B**). These conditions help metastatic cancer cells floating in the peritoneal fluid overcome anoikis (15, 35, 36).

The peritoneal cavity is lined by a single layer of mesothelial cells, known as the mesothelium, which covers all visceral organs. The peritoneal mesothelium not only provides a non-adhesive protective surface to facilitate the movement of organs within the cavity but also serves as an important immune barrier against breaching of microorganisms (37). During ovarian cancer metastasis, mesothelial cells stimulated by cancer cell- or macrophage-derived cytokines, such as transforming growth factor beta 1 (TGF-β1) and macrophage inflammatory protein 1 beta (MIP-1β), promote cancer cell adhesion, invasion, and proliferation via expression of fibronectin, stromal cell-derived factor-1α (SDF-1α) and P-selectin (38–40). Mesothelial cells also support cancer cells by undergoing mesothelial-to-mesenchymal transition (MMT) induced by cytokines such as TGF-β, hepatocyte growth factor (HGF), and plasminogen activator inhibitor-1 (PAI-1) from cancer cells (41–43). In addition, spheroids in contact with mesothelial cells facilitate their migration and clearance, allowing cancer cells access to the sub-mesothelial environment (44) (**Figure 1C**).

The omentum is a central regulator of intraperitoneal homeostasis, controlling inflammation, regulating fluid exchange, promoting angiogenesis, and storing and supplying lipids (45). Underneath the mesothelial cells that cover the surface are abundant adipocytes, adipose-derived stromal cells (ASCs), fibroblasts, and immune cells (14). Milky spots are lymphoid tissues within the omentum consisting mainly of macrophages, T cells, and B cells. Importantly, the mesothelial lining in the milky spots is not continuous, which enables circulating leukocytes to migrate into the peritoneal cavity (46). Milky spots are also known to be a major implantation site of cancer cell metastasis (47). Indeed, cancer cells preferentially lodge and grow in omental milky spots rather than in other peritoneal fat depots (48). Macrophages in the milky spots promote colonization of cancer cells via secretion of C-C motif chemokine receptor 1 (CCR1) ligand (49). It was also reported that neutrophil influx to the omentum, predominantly in milky spots and through neutrophil extracellular traps (NETs), facilitated a premetastatic niche (50). In addition, a mechanism of hematogenous metastasis with a preference for the omentum via the ErbB3-neuregulin1 axis has been reported (51). Omental adipocytes increase adipokine secretion, such as Interleukin 8 (IL-8) and C-C motif chemokine ligand 2 (CCL2) to promote homing of ovarian cancer cells to the omentum (52, 53). Moreover, once cancer cells are seeded in the omentum, adipocytes transfer fatty acids to cancer cells and increases energy production through fatty acid oxidation (53, 54). ASCs in the omentum increase production of nitric oxide (NO) and matrix metalloproteinases (MMPs) in ovarian cancer cells and promote cancer cell growth and metastasis (55, 56). Finally, ovarian cancer cells can also transform omental fibroblasts into cancer associated fibroblasts (CAFs) via miRNAs and TGF-β1 to modulate the tumor microenvironment at the metastatic niche (57, 58) (**Figure 1D**).

In summary, there are supportive mechanisms for ovarian cancer that are specific to each of the organs of the peritoneal fluid, peritoneum, and omentum.

## Identification and characterization of tissue-resident macrophages in the peritoneal cavity in mice

Peritoneal fluid contains an abundance of immune cells, of which macrophages are the most dominant, accounting for about half of the total number (59). In the steady state, macrophages do not adhere to the peritoneum but float in the peritoneal fluid (59). Fate mapping studies have shown that there are two types of resident macrophages in the peritoneal fluid in mice: Large peritoneal macrophages (LPMs) from embryonic origins and small peritoneal macrophages (SPMs) derived from circulating monocytes (22). LPMs represent approximately 90% of intraperitoneal macrophages at homeostasis and are characterized by the expression of GATA6 and GATA6-regulated peritoneal macrophage-specific genes (20, 21, 60). Retinoic acid supplied by omental adipose tissue and Wilms tumor 1 (WT1)-expressing mesothelial cells or submesothelial fibroblasts plays a central role in GATA6 expression in LPMs (60, 61). Although the composition of SPM niche is unclear, development of SPMs requires IRF4 and signals from microbiome (62). LPMs express a higher level of F4/80 and lower level of MHCII than SPMs (20), however, intracellular adhesion molecule 2 (ICAM2) and Tim-4 are specifically expressed by LPMs and CD226 in SPMs, therefore they are considered selective markers (62–65). Embryo-derived LPMs are partially replaced by monocyte-derived macrophages during aging and inflammation (66–68). Interestingly, monocyte-derived macrophages that replace the resident LPMs after inflammation exhibit a phenotype similar to embryo-derived LPMs (69, 70). LPMs phagocytose more and differentially secrete inflammatory cytokines in response to stimuli when compared to SPMs (71). Moreover, LPMs rapidly form multicellular aggregates at the site of intraperitoneal bacterial exposure or peritoneal injury to control the spread of bacterial infection and assist in wound healing respectively (59, 72, 73). In addition, LPMs are known to be involved in IgA production by B1 cells in gut-associated lymphoid tissue (GALT) (60) and play an important role in B1 cell homeostasis.

In addition to resident macrophages in the peritoneal fluid, macrophages within parietal membranes in mice have been described by Uderhardt et al. (74). Specifically, a resident macrophage population was identified in mesothelial cell layers of the mesentery and peritoneum, characterized by CD64^+^F4/80^+^LYVE1^hi^ expression, and noted to be distinct from macrophages in the peritoneal fluid (24). These LYVE1^hi^ membrane-associated macrophages derive primarily from embryonic progenitor cells and are regulated by colony stimulating factor 1 (CSF1), which is produced by WT1^+^ stromal cells. These macrophages resemble LYVE1^+^ macrophages present on the surface membranes of other organs (with the exception on the liver) and have a different gene expression phenotype from resident macrophages in other body compartments, such as peritoneal fluid macrophages, alveolar macrophages, and microglia (24).

In the murine omentum, there is also a dominant LYVE1^+^ macrophage population. Among the LYVE1^+^ macrophages (also CD169^+^), the CD163^+^Tim-4^+^ population has been shown to be the resident fraction derived from embryonic progenitor cells, while others derive from monocytes. This embryo-derived population is found in the vicinity of the milky spots and has a unique gene expression pattern that is enriched in the JAK-STAT pathway (25). LYVE1^+^ macrophages in the omentum during the embryonic period have been shown to regulate lymphatic permeability and function through modulation of IL-1β production (75).

**Figure 2:**
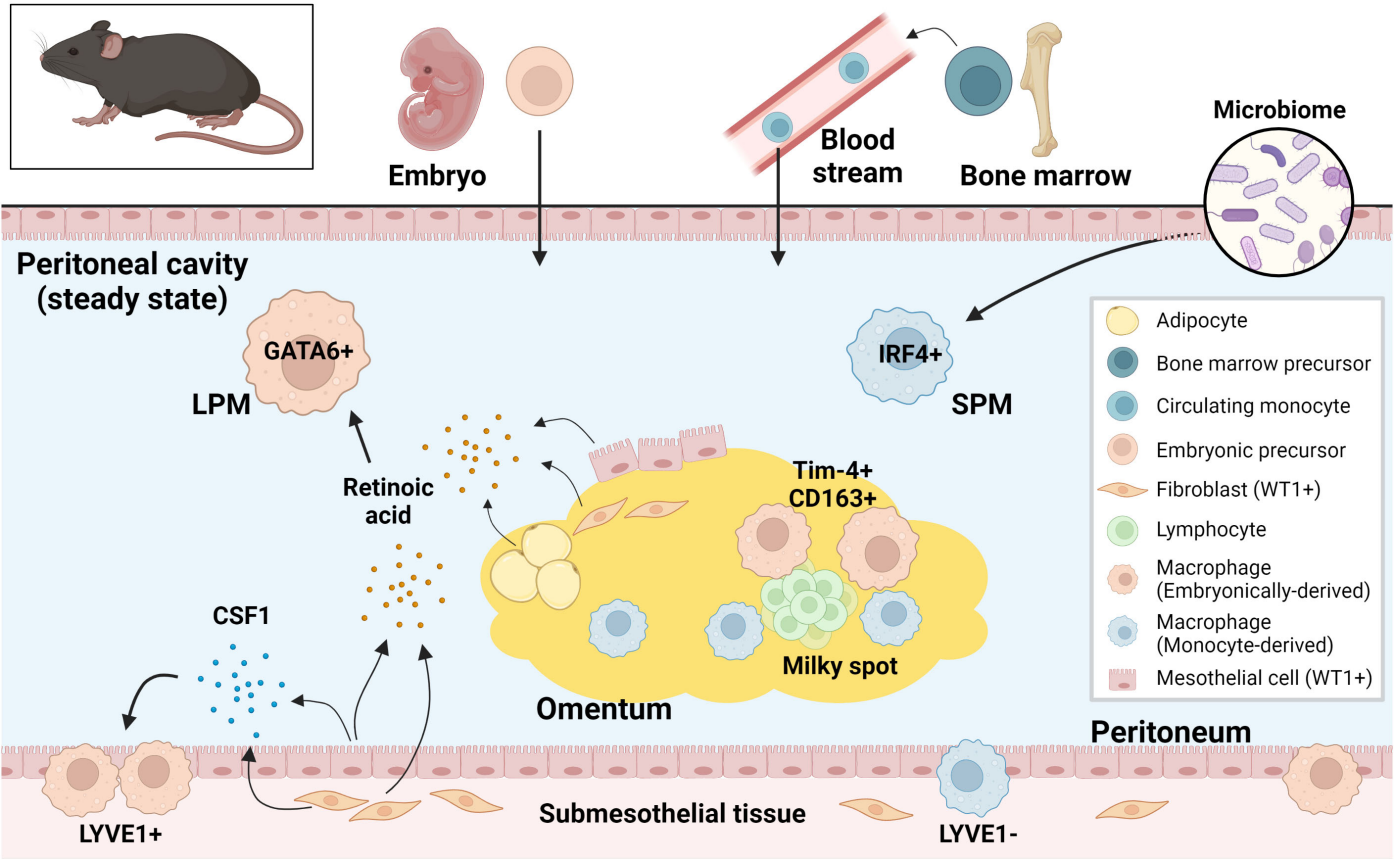
Murine resident peritoneal macrophages and their supporting environment at steady state. Both embryonically-derived and monocyte-derived resident macrophages are present in murine peritoneal cavity at steady state. These resident macrophages from different origins are regulated differently in the tissue-specific niche. LPM, large peritoneal macrophage; SPM, small peritoneal macrophage; CSF1, colony stimulating factor 1; WT1, Wilms tumor 1.

**Figure 3:**
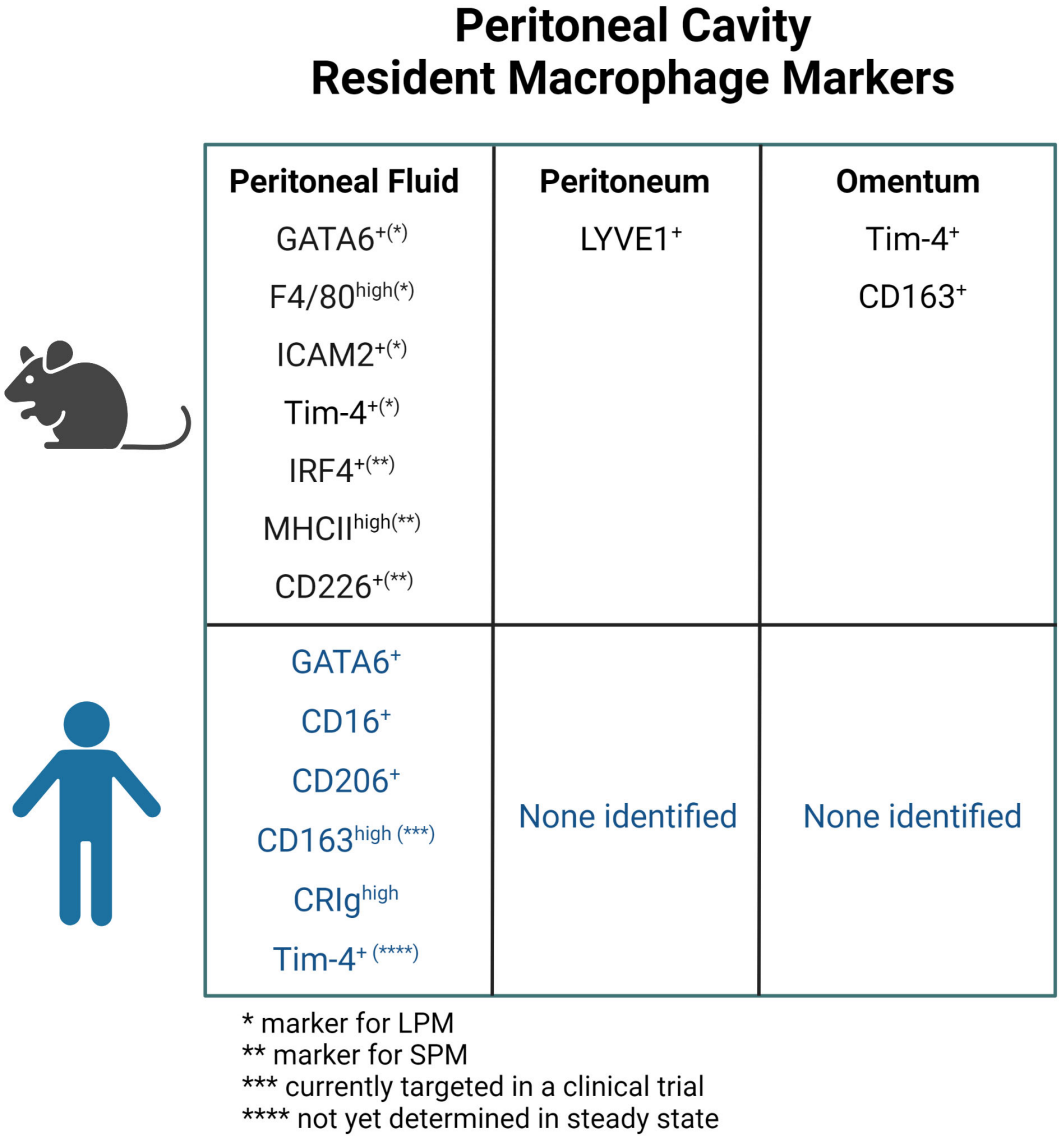
Markers for resident macrophage in the peritoneal fluid, peritoneum, and omentum in mice and human. ICAM, intracellular adhesion molecule; LPM, large peritoneal macrophage; SPM, small peritoneal macrophage.

Thus, tissue-specific resident macrophage fractions of embryonic or monocyte origins have been identified in the peritoneal fluid, peritoneum and omentum, revealing the origin- and compartment-based diversity of macrophages in the peritoneal cavity (**Figure 2**). Markers for compartment-specific resident macrophage in the peritoneal cavity are summarized in **Figure 3**.

## The role of resident peritoneal macrophages in ovarian cancer progression in mice

Macrophages in the peritoneal cavity contribute to the intraperitoneal metastasis of ovarian cancer through spheroid formation, increased adhesion of cancer cells to mesothelial cells, and colonization to milky spots in omentum (36, 40, 49). Macrophage-mediated inflammation has also been shown to be important in the progression of ovarian cancer (76). In the past decade, it has been elucidated that macrophages of both embryonic and monocytic origins coexist in tumors in varying proportions, and that these different macrophages have distinct effects on tumorigenesis depending on tumor types (27).

First, regarding macrophages in the peritoneal fluid, Xia et al. showed that in an ID8 ovarian cancer model, macrophages in the peritoneal fluid can be divided by Tim-4 expression into the Tim-4^+^GATA6^+^ resident type and the Tim-4^-^GATA6^-^ monocyte-derived type. Functionally, monocyte-derived macrophages do not contribute to ID8 tumorigenesis using CCR2 deficient murine models, whereas resident peritoneal macrophages support tumor progression. These protumor resident macrophages have high arginase 1 expression, high mitophagy activity, and decreased mTORC1 activity. Selective autophagy deficiency in myeloid cells by deleting FIP200 induces loss of Tim-4^+^ macrophages, enhanced T cell immunity, and suppressed ID8 intraperitoneal tumor growth *in vivo* (28). Casanova-Acebes et al. showed that Tim-4^+^GATA6^+^ LPMs are maintained by retinoid X receptors (RXRs). RXR deficiency 1.) reduces the survival of Tim-4^+^ LPMs through excess lipid accumulation, 2.) reduces LPM accumulation in early ovarian tumors, and 3.) slows primary ovarian tumor growth (65). Weiss et al. showed that the immune responsive gene 1 (IRG1)-synthesized metabolite, itaconic acid, and oxidative phosphorylation are upregulated in F4/80^+^ resident macrophages in intraperitoneal tumors. Interestingly, IRG1 deletion in macrophages reduces oxidative phosphorylation and subsequent reactive oxygen species (ROS) production in resident macrophages, which leads to attenuated ROS-mediated MAPK activation in tumor cells and suppressed growth of ID8 intraperitoneal tumors (77).

Embryonic LYVE1^+^ resident macrophages in the peritoneum are characterized by increased expression of alternatively activated macrophage genes such as *Retnla* (RELMα) and *Mrc1* (CD206). Although LYVE1^+^ macrophages are also present in the omentum, removal of LYVE1^+^ macrophages suppressed intraperitoneal tumor growth of ID8 cells in an omentectomized mouse model, demonstrating that LYVE1^+^ macrophages in the peritoneal membrane promote intraperitoneal expansion of ovarian cancer independent of the omentum (24).

Etzerodt et al. showed that resection of the omentum prior to tumor cell injection delayed intraperitoneal progression in the ID8 mouse model of ovarian cancer, confirming the tumor-promoting properties of the omentum in ovarian cancer. They showed that Tim-4^+^CD163^+^ resident macrophages in the omentum contributed to the acquisition of a cancer stem cell (CSC)-like phenotype in tumor cells and increased the tumor cell number in the ascites without affecting tumor development in the omentum. Furthermore, continuous administration of CD163-targeted lipid nanoparticles (LNPs) specifically reduced the numbers of embryonic Tim-4^+^CD163^+^ and monocyte-derived Tim-4^-^CD163^+^ macrophages. This led to reduced tumor burden in both the omentum and ascites (25). These data indicate that macrophage subpopulations in the omentum may be separately involved in tumor progression. However, the mouse models used in this study cannot separate the role of omental macrophages from membrane macrophages, because both omental and membrane but not fluid macrophages express CD163 (24, 25). Further studies need to design a model that can specifically target the omental macrophage.

In summary, embryonically-derived resident macrophages in the peritoneal fluid, peritoneum, and omentum promote intraperitoneal progression of ovarian cancer. Tim-4, LYVE-1, and CD163 are some of the surface markers for the resident macrophages in the peritoneal fluid, membrane, and omentum, respectively. It remains less well understood what roles monocyte-derived macrophages play in the progression of ovarian cancer in these peritoneal compartments.

## Resident peritoneal macrophages and ovarian cancer in humans

Unlike murine resident macrophages, resident peritoneal macrophages in humans are much less well-studied, mainly due to the limited sample availability, particularly in the steady state. Furthermore, it is nearly impossible to definitively determine their precise origin because of ethical concerns. Therefore, in this review, we consider human macrophages as tissue resident when they are present before detectable diseases. Whether they develop prenatally during embryogenesis or postnatally from circulating monocytes remains unknown and difficult to test. Despite such limitations, recent technical advances have led to important discoveries of some aspects of resident peritoneal macrophages in humans.

Like in mice, macrophages are the predominant immune cell component in human peritoneal fluid (78). Although it remains controversial whether resident macrophages in human peritoneal fluid require GATA6 for survival and self-renewal, Ruiz-Alcaraz et al. analyzed peritoneal fluid from patients undergoing tubal ligation or gynecological surgery for benign tumors and found that over 80% of CD14^+^CD16^+^ cells in the monocyte/macrophage fraction in humans were GATA6 positive (79). On the other hand, CD14^+^ macrophages in cancerous ascites did not appear to express GATA6 (80). Interestingly, resident macrophages are decreased in cancer tissues compared to normal tissues in lung cancer (81), which could potentially explain the difference in GATA6 expression in the peritoneal macrophages between healthy donors and cancer patients (67). Interestingly, the analysis of peritoneal fluid from infants to adolescents who underwent surgical procedures that were considered immunologically intact showed that the percentage of CD14^+^CD16^high^ fractions was higher in infants and lower in adolescent children, which might reflect age-related changes in resident macrophages (82). This is somewhat consistent with the murine data showing that increasing proportions of resident peritoneal macrophages derive from monocyte precursors in the bone marrow as mice age (68).

Two studies have suggested CD16^+^CD206^+^ macrophages as resident macrophages in the peritoneal fluid in humans. In one study, a CD16^+^CD206^+^ macrophage fraction can be detected in patients before peritoneal dialysis, whose peritoneal environment is considered as homeostatic and physiologic. These CD16^+^CD206^+^ “mature” macrophages were abundant before dialysis, but decreased after dialysis, whereas the CD16^+^CD206^-^ fraction increased after dialysis. Moreover, during acute peritonitis, the CD16^+^CD206^+^ macrophage fraction decreased and the CD16^-^CD206^-^ fraction increased compared to the steady state, which is consistent with a decrease in the resident macrophage fraction during infection in mice (83). In the other study, Stengel et al. characterized macrophages in ascites from cirrhotic patients by CD206 expression and defined CD206^+^ cells as human LPMs and CD206^-^ cells as human SPMs because the former was larger and more granular. Human LPMs contain a homogenous CD16^+^ and CCR2^-^ population and express higher levels of CD163 and CRIg than human SPMs. Additionally, human LPMs showed higher Ki-67 positivity than human SPMs, which indicates the increased proliferative potential of human LPMs. Moreover, the proportion of human LPMs decreases in the presence of peritoneal infection, which phenocopies what has been reported in mice LPMs (84). Irvine et al. compared macrophages in human cirrhotic ascites with mouse peritoneal fluid macrophages by RNA sequencing. They showed similarities between CRIg^high^ human macrophages and F4/80^high^ murine resident macrophages, as well as between CRIg^low^ human macrophages and monocytes and F4/80^low^ murine monocyte-derived macrophages. These hypothesize that CRIg^high^ and CRIg^low^ macrophages may represent tissue-resident and monocyte-derived populations in humans, respectively (85). Importantly, recent single cell RNA sequence data confirmed expression of CD16 (*FCGR3A*), CD206 (*MRC1*), *CD163*, and CRIg *(VSIG4*) in macrophages from both peritoneal fluid at steady state and ovarian cancer ascites (33, 86).

Tim-4 is another potential marker for resident peritoneal macrophages in humans. In ovarian cancer ascites from patients, Chow et al. demonstrated the presence of Tim-4^+^ macrophages (which marks tissue-resident macrophages in murine peritoneal fluid), but Tim-4 was not expressed on steady-state circulating monocytes. Moreover, Tim-4 expression within tumor tissues was detected only in the remaining native tissue compartment, not in the tumor-invaded area, suggesting that Tim-4 is also a specific marker for resident macrophages in human peritoneal fluid (80). Another study by Xia et al. reported only 3% of Tim-4^+^ macrophages in ovarian cancer ascites. They postulated that Tim-4 may not be a marker for the resident macrophage fraction in ovarian cancer ascites, but they also acknowledged the possibility of poor sensitivity of the anti-human Tim-4 antibody they used. Using RNA seq data of macrophages in ovarian cancer ascites (87), they showed a similarity in ovarian cancer ascites between human CRIg^high^ macrophages and murine Tim-4^+^ macrophages, and that ovarian cancer patients with higher CRIg^high^ expression had poor prognosis (28).

Even less is known about resident macrophages in the peritoneal membrane and omentum in humans. Single-cell RNA seq of metastatic omental tumor of ovarian cancer identified a CD163^+^CD204^+^ cluster with high CD14 and CD16 expression and a NR1H2^+^ cluster in macrophages of all 6 cases (88). This may indicate the presence of specific macrophage subsets in the omentum. However, it is not clear whether these macrophage subsets are also present in the steady state, i.e., resident macrophages.

Thus, it has been suggested that molecules such as CD16, CD206, CD163, CRIg, and Tim-4 may be associated with resident macrophages in the human peritoneal fluid, and macrophages expressing these molecules are also found in ascites from ovarian cancer patients (**Figure 3**).

## Therapeutic potential of resident peritoneal macrophages for ovarian cancer

There has been a significant development of therapies targeting macrophages in the tumor microenvironment. The two main approaches are 1.) reducing the number of macrophages, via CSF1-colony stimulating factor 1 receptor (CSF1R) or CCL2-CCR2 pathway inhibition, and 2.) exploiting the macrophage plasticity by reprogramming immunosuppressive macrophages to immunoreactive, as represented by successful targeting the CD47-SIRPα pathway (30). However, apart from toll-like receptor (TLR) agonists, such as imiquimod and BCG, most of the therapeutic strategies that showed efficacy in preclinical models have yet to become the standard treatment in clinical settings (89). Clinical efficacies of macrophage-targeting therapies in solid tumors have been limited due to the complexity of the origin, plasticity, and intra-tumor heterogeneity of macrophages (27, 29, 30). Recently, adoptive immunotherapy using genetically modified macrophages (chimeric antigen receptor-macrophage; CAR-M) with enhanced phagocytosis and high T cell costimulatory capability has been developed. A Phase I trial using CAR-M that recognize HER2 antigen have been initiated to verify its safety and efficacy (29, 90, 91).

The contribution of resident macrophages to tumor progression has been demonstrated not only in the peritoneal cavity, but also in the brain (92), pancreas (93), and lung (81, 94), suggesting the promising therapeutic potential of targeting resident macrophages in cancer. Below, we highlight strategies of targeting several potential markers of resident peritoneal macrophages as cancer therapies. Because resident macrophages in the omentum and the peritoneal membrane were only recently characterized in tumor models, there has been no published data on specifically targeting these two populations. The following summary only focuses on resident macrophages in the peritoneal fluid.

As previously discussed, Tim-4 is a phosphatidylserine receptor that has been identified in resident peritoneal macrophage in both mice and humans (28, 80, 95). In a murine intraperitoneal metastatic model of colorectal and lung cancer, Tim-4 blockade alone showed no significant effect on tumor progression. However, combined with an immune checkpoint inhibitor, Tim-4 blockade significantly slowed tumor progression, proposing the treatment strategy that blockade of Tim-4-mediated sequestration against phosphatidylserine^+^CD8^+^ T cells to enhance the efficacy of CD8^+^ T cell-based immunotherapies (80). Interestingly, targeting another phosphatidylserine receptor, MerTK, which is in the same pathway (efferocytosis) as Tim-4, in these macrophages increases anti-tumor immunity (96), suggesting that targeting the efferocytosis pathway in resident peritoneal macrophages holds a great promise in treating intraperitoneal metastasis of ovarian cancer.

Another potential cross-species marker of resident peritoneal macrophage is CRIg, also known as VSIG4. It has been characterized as a complement receptor as well as a co-inhibitory immune checkpoint molecule of T cells (97, 98). An exciting ongoing study highlights an anti-CRIg antibody (VTX-1218) by Verseau Therapeutics. Preliminary results suggest a synergistic anti-cancer effect of VTX-1218 and immune checkpoint therapy in syngeneic mouse models. Mechanistically, VTX-1218 repolarizes tumor-associated macrophages into proinflammatory phenotypes and promotes T cell-mediated tumor killing (99). Further studies using VTX-1218 or targeting CRIg in murine models of ovarian cancer is needed to validate these preclinical data targeting this pathway.

CD206 and CD163 are other candidates to target on resident peritoneal macrophages in humans. Because both molecules have garnered attention as a marker of immunosuppressive M2 macrophages within TAMs, therapeutic development targeting them is being vigorously pursued (100, 101). However, it should be noted that those molecules can also be expressed on TAMs differentiated from circulating monocytes and therefore therapies targeting them may not be specific to tissue-resident fraction in cancer setting. For example, CD206 expression can be induced on CD14^+^ monocytes from peripheral blood cocultured with IL-6, one of the major cytokines upregulated in ovarian cancer ascites (102, 103). Nonetheless, Zhou et al. showed that Fe_3_O_4_-based polylactic-glycolic acid (PLGA) nanoparticles, whose surface was modified with an anti-CD206 monoclonal antibody, repolarized M2 macrophages to a M1 phenotype (104). Additionally, Jaynes et al. developed a 10-mer peptide (RP-182) that selectively induces a conformational switch of CD206 from the open to the closed state. This activation enhances endocytosis, phagosome-lysosome formation, and autophagy programs, resulting in reprogramming M2-like tumor associated macrophages to an antitumor M1-like phenotype. RP-182 suppresses tumor growth in a mouse pancreatic cancer model and synergizes with chemotherapy (105). Finally, a bispecific T-cell engager (BiTE) recognizing CD206/CD3 and a trispecific T-cell engager (TriTE) with bivalent anti-CD3 binding have also been developed. These BiTEs and TriTEs can activate T cells and induced cytotoxicity toward M2 macrophages *in vitro* (106). Etzerodt et al. demonstrated that depletion of CD163^+^ macrophages with doxorubicin-loaded, antibody-conjugated lipid nanoparticles inhibited tumor growth in an intraperitoneal metastasis model of melanoma (107). Moreover, OR2805, an anti-CD163 antibody, has been developed and is now in phase I/II clinical trials as a single agent or in combination with a PD-1 antibody against multiple tumor types (NCT05094804). Preliminary results demonstrate an anti-tumor activity in lung cancer xenograft models in humanized mice (108).

In summary, therapies targeting molecules that are regarded as markers of resident macrophages are being eagerly developed. In particular, an anti-CD163 antibody is being tested in a phase I/II clinical trial.

## Future challenges about resident peritoneal macrophages in relation to ovarian cancer

During intraperitoneal metastasis of ovarian cancer, it is still not clear how much of the intra-tumoral macrophages are derived from the resident fraction, or how plastic they are (i.e., whether they switch between M1/M2 phenotypes). Although macrophages derived from circulating monocytes are generally considered to be highly plastic, tissue-resident macrophages appear to have a restricted plasticity (26). This may be because it is desirable to limit the plasticity of resident macrophages, which remain in tissues for a long time and whose main function is to maintain tissue homeostasis (26). Indeed, in lung cancer, resident macrophages and monocyte-derived macrophages coexist in early lung cancer lesions and retain their distinct phenotypic and molecular programs even in late-stage tumors (81). Therefore, reprograming resident macrophages within human tumors may pose a significant challenge. From this perspective, depletion might be better way than repolarization for targeting resident macrophage.

A lack of understanding resident peritoneal macrophages is another hurdle against the therapeutic development. All murine resident macrophages in the peritoneal cavity (fluid, membrane, omentum) act in a tumor-promoting manner during ovarian cancer metastasis, but the detailed mechanisms of how they contribute to tumor progression is still not fully elucidated. In addition, resident macrophages in the ovary and fallopian tubes have not been studied in detail, and the impact of these resident macrophages on primary tumor development is unknown. Finally, comprehensive characterization of resident macrophages in the human peritoneal cavity has only just begun to emerge. Specific transcriptional regulatory mechanisms modulated by the unique intraperitoneal environment (such as retinoic acid and GATA6 in murine LPMs) have not been identified in humans. Therefore, attempts to translate from preclinical mouse models to human therapies are significantly impinged. Although it remains difficult to collect steady-state samples, it is necessary to continuously attempt a comprehensive analysis of tissue macrophages, including in non-cancerous environments, when approaching the peritoneal cavity during benign or malignant surgeries.

Current therapies for late-stage ovarian cancer patients mainly rely on intravenous chemotherapy. However, the unique microenvironment of the peritoneal cavity (highly dynamic fluidics, crosstalk between multiple tissue compartments, and the peritoneal-plasma barrier) prompts the field to consider shifting to an intraperitoneal delivery (109). Although intraperitoneal chemotherapies show inconsistent results in ovarian cancer patients, they have shown a tremendous improvement in patients with intraperitoneal metastasis of other cancers (109). In order to efficiently and specifically target resident peritoneal macrophages, intraperitoneal injection is also a preferred option in comparison to intravenous injection. Future macrophage-based therapies need to comprehensively compare these two routes of drug delivery in preclinical models and clinical settings.

## Concluding remarks

The peritoneal cavity is a dynamic microenvironment with a wide variety of functions that interact closely with all peritoneal tissues. Only recently have resident macrophages in the peritoneal cavity been identified and their pro-tumor roles in ovarian cancer metastasis has come under scrutiny. Because most ovarian cancer growth and metastasis occur in the peritoneal cavity, a better understanding of the unique microenvironment and cellular characteristics present in the peritoneal cavity is essential for combating ovarian cancer metastasis. Besides the well-established T cell-based immune therapies, we call for more extensive research on macrophage-targeting therapies, which may lead to a cure for ovarian cancer in combination with other established immune therapies and targeted therapies.

## Author contributions

All authors listed have made a substantial, direct, and intellectual contribution to the work and approved it for publication.
